# Reduced-Exertion High-Intensity Interval Training as a Novel Therapeutic Approach for Insomnia

**DOI:** 10.3389/fpsyt.2021.754171

**Published:** 2021-10-29

**Authors:** Hady Atef, Taulant Muka

**Affiliations:** ^1^Faculty of Physical Therapy, Cairo University, Giza, Egypt; ^2^Department of Preclinical Medicine, Medical School, Institute of Social and Preventive Medicine (ISPM), University of Bern, Bern, Switzerland

**Keywords:** insomnia, alternative and complementary therapy, reduced exertion high intensity training, exercise, cardiorespiratory fitness

Sleep deprivation, and decline in cardiorespiratory fitness are among many of the mortality risks found among different sleep and cardiorespiratory conditions. Control of these mortality risks may not only improve the condition-but in some times- may reverse the underlying disorder. Also, sleep deprivation can lead to a spectrum of negative clinical consequences including suicidal attempts (1). Additionally, data from the prospective population studies are consistent in showing that both short and long duration of sleep are predictors of mortality. There are bidirectional interactions between sleep and exercise, both affect each other through multiple physiological and psychological pathways, having further a role on mortality (2, 3).

Insomnia in general has two main subcategories: primary and secondary types (1, 2). Table 1 summarizes the main differences between them.

**Table 1:** Main characteristics of primary and secondary insomnia.

**Primary insomnia**	**Secondary insomnia**
It is an inherent sleep dysfunction which is not affected by any environmental factors; therefore, it is difficult to grasp and treat.	It is an acquired condition which is affected by external factors (pain, anxiety…etc.); therefore, it is much easier to grasp and treat.
Genetic predisposition is the predominant factor leading to this	Many factors can be triggering to this kind of insomnia like stress, medications, sleep disorders…etc.
Less common	More common
It frequently leads to paradoxical insomnia and mental health affection	Mostly reversible but would lead to serious effects like depression, anxiety, and cardiovascular complications if persisted for a long time

Importantly, in a growing number of large-scale studies, cardiorespiratory fitness (CRF) is shown to be a more powerful predictor of risk for many cardiorespiratory disorders and all-cause mortality than more commonly used risk factors such as smoking, hypertension, obesity, high cholesterol, and insulin resistance (4).

Although exercise is the only viable means of improving CRF, and can also prevent or treat hypertension, obesity, high cholesterol, and insulin resistance, it remains underutilized globally as a means of primary disease prevention and treatment (5).

Exercise is a major burden for many cardiac and chest pain patients, the current advised exercises and rehabilitation protocols may be very intense for some of the patients and unrealistic options, decreasing patient's adherence to the treatment. Thus, identifying alternative approaches that may improve exercise tolerability and acceptability in these cases is desirable. “Reduced exertion interval training” (REHIT) is a novel exercise usually done on a special bike, characterized by being short, non-exhausting, self-paced and time-efficient with proven superior effect over the traditional exercises in different clinical populations, but its role in sleep and cardiorespiratory disorders remains to be elucidated from future clinical studies (4).

## Why REHIT?

**Figure 1:**
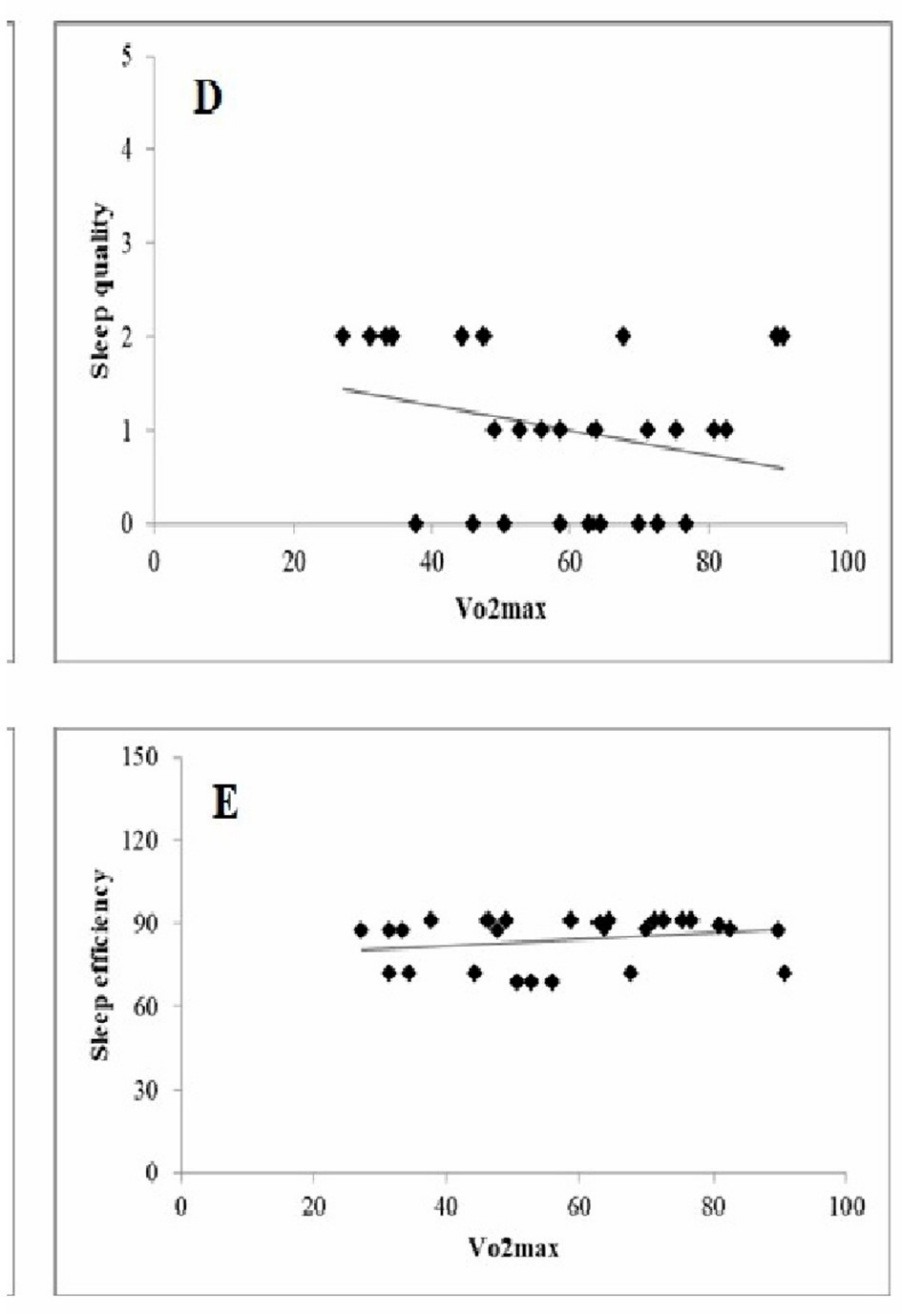
Simple correlation between (sleep quality, sleep efficiency), and VO2max (cardiorespiratory fitness).

From literature, we found that there is evidence of an association between CRF and sleep outcomes. Both affect each other in different populations, and the greatest morbidity and mortality risk is observed in subjects with both sleep deprivation and CRF decline. To date, the REHIT is the shortest duration of high-intensity exercise with actual demonstrated health benefits, which includes two 20-s all-out cycle sprints in a 10-min low-intensity exercise session. Six weeks of REHIT training 3 times/week improved VO2max in sedentary subjects with cardiovascular risk, as well as insulin sensitivity. It was proved that REHIT can improve CRF better than the traditional exercise options, and with less time commitment. Consequently, we suggest its superior effect on insomnia; keeping in mind the positive correlation between insomnia and the CRF. When it came to the subject's perception of the REHIT, it was positive, and the subjects recommended its use over the traditional continuous exercise; being more enjoyable and time-efficient. All of the above-mentioned reasons would support the REHIT use for insomnia, and would suggest its superior effect over the available exercise options on sleep deprivation (Figure 1) (5–7).

### Exercise Description

Participants doing REHIT are usually asked to perform 2 exercise sessions per week. Each session involves 10 min of low-intensity cycling (25 W) interspersed with two 20-s “all-out” cycle sprints against a resistance equivalent to 5% of body mass. The exercise intervention is delivered on a cycle ergometer, and does not require no support for delivery. The location of the equipment can be flexible, with the ideal location likely close to the rehabilitation department of the hospital. A computer screen on the handlebars provides guidance and feedback throughout each training session, with an option for headphones to receive the information in audio-format. Power output and heart rate (measured through contact of the hands with the handlebars) during the sprints will be recorded for each session. Notably, as participants need to log in on the bike's screen with a personal code, adherence is monitored, regular individual feedback can be provided, and poor compliance will trigger email reminders (4, 8, 9).

The strength of REHIT would be to introduce a very short and non-exhausting form of exercise that lasts for 10 min in total, with an estimated superior effect than the available long duration exercises, thus, increasing patient commitment; and improving patient-centered outcomes (PCO). In pediatrics, also many children, who are recommended for an active lifestyle, are not committing to the recommended doses of physical activity, in some way because the current available exercise recommendations fail to address important exercise restrictions within this group. REHIT might also be helpful in children who are indicated for the rehabilitation programs, as it offers an opportunity to overcome several common perceived barriers to exercise within this group of patients. REHIT (1) improves CRF more than traditional aerobics (2) is manageable, well-tolerated, and acceptable by the patients (3) individually tailored, so the child will not be in any challenging situation, and (4) can be done on a special bike, in an amusing form and different entertaining method to avoid boredom experienced by most of the children in traditional cardiac rehabilitation programs. REHIT is much more entertaining than traditional aerobic exercises (4, 5).

We present this viewpoint in highlighting the potential benefits of REHIT over the traditional available exercise options, with the promise that could be used more in the clinical work in the future. A better grasp of the REHIT mechanisms and insomnia pathologies through future RCT studies may play a key role in understanding the association between REHIT and insomnia, and its benefit in the clinical practice. To fully understand the expected effect of the REHIT on insomnia and why we propose REHIT in specific, we must understand more information than given by the mechanism of action of the REHIT and investigate the evolutionary origins of that exercise (5, 10).

These potential benefits were in line with the results reported by Gillen et al. (11) who utilized almost a similar methodology but with three cycles of 20-s sprints instead of two. Similar improvements in VO2 max compared to Metcalfe study were associated with parallel aerobic adaptations in skeletal muscle (increased protein content of COX IV and increased citrate synthase maximal activities). Consequently, we can consider the REHIT as the most available exercise option which is highly time-saving (the training period is only 30 min per week) and not exhausting option (“somewhat hard”), and conclude that it may provide a feasible, acceptable, and manageable exercise option or an add-on to the current physical activity/sleep physical interventions recommendations. However, future larger RCTs will be needed to confirm these findings (4, 5, 10).

Cardiovascular (CV) outcomes are many, including resting and dynamic ones. Cardiopulmonary (CP) outcomes include different outcomes, but CRF is the most important one being the most important mortality factor within these cardiopulmonary outcomes. In previous articles, we discussed how the management of the CV and CP outcomes that occur through physical training assists in restoring the optimal structure of sleep. This synchronization is directly associated with an increase in sleep quality and quantity. Such interaction is bi-directional as CV and CP outcomes are strongly influenced by the autonomic nervous system signals. We support the opinion that voluntarily fostering CV and CP outcomes can influence the autonomic nervous system toward the dominance of the parasympathetic tone and may restore the normality of the sleep architecture. Exercise has been shown to promote a high degree of improvement in CV and CP with a special focus on CRF. Also, exercise has a demonstrated effect on sleep directly with many physiological explanations. It has been shown that stress is inversely correlated with CV and CP outcomes and decreased sympathetic tone. Therefore, through the techniques that allow for the modulation of these CV and CP outcomes like physical training, we suggest one may better manage stress, CV and CP outcomes; and consequently sleep (12–14).

Regarding the limitations of the REHIT, first, it has to be applied on a special bike, however being relatively cheap and commercially available in the European and American market, this can be a methodological barrier in some low-income countries' studies. Furthermore, however being an individually-tailored exercise, it is a challenging form of it; that may be injurious in some instances if the subject overstrain himself/herself and doesn't follow the instructions given by the trainer/therapist, so supervision is sometimes recommended to limit these adverse effects.

In conclusion, the new REHIT may positively affect cardiopulmonary, cardiovascular outcomes, stress, skeletal muscle relaxation accompanied by improvement in cardiorespiratory fitness (which is directly proportional to all sleep outcomes). We have suggested that modulation of the CV, and CP systems via exercise like REHIT in adjunct/instead of traditional exercise options, together with techniques like sleep hygiene, may be a feasible and acceptable tool in controlling insomnia than the known methods of using aerobics, resisted exercise, and other pharmaceutical interventions like agomelatine therapy which is frequently used for the management of insomnia in different psychiatric conditions like the acute bipolar II depression (15). Concerning the close association between the CV, CP, sleep physiology, and respiration state discussed, it is clear that it is possible to improve sleep architecture voluntarily, through engaging in physical interventions such as the proposed REHIT. We have proposed that REHIT and other sleep hygiene methods may be a superior option for a better activation of the VO2max than the traditional exercise options, in a help for people to deal with the insomnia. Literature has significant evidence for the efficacy of relaxation and sleep hygiene techniques in treating insomnia, however, according to our knowledge, no research investigating treatment with REHIT. Through our viewpoint, we hope to inspire discussion, debate, and future research into REHIT as a novel, optimistic, and a potential effective exercise option for insomnia; having a superior effect on CRF than the traditional exercise options. Also, to compare between the effect of the REHIT on the structure of sleep in general with the traditional exercise usually utilized for improving sleep quality and quantity.

## Author Contributions

All authors listed have made a substantial, direct and intellectual contribution to the work, and approved it for publication.

## Conflict of Interest

The authors declare that the research was conducted in the absence of any commercial or financial relationships that could be construed as a potential conflict of interest.

## Publisher's Note

All claims expressed in this article are solely those of the authors and do not necessarily represent those of their affiliated organizations, or those of the publisher, the editors and the reviewers. Any product that may be evaluated in this article, or claim that may be made by its manufacturer, is not guaranteed or endorsed by the publisher.

## References

[1] De Berardis D Fornaro M Valchera A Cavuto M Perna G Di Nicola M. Eradicating suicide at its roots: Preclinical bases and clinical evidence of the efficacy of ketamine in the treatment of suicidal behaviors. Int J Mol Sci. (2018) 19:2888. 10.3390/ijms19102888 30249029 PMC6213585

[2] Yang P Ho K Chen H Chien M. Exercise training improves sleep quality in middle-aged and older adults with sleep problems : a systematic review. J Physiother. (2012) 58:157–63. 10.1016/S1836-9553(12)70106-6 22884182

[3] Kovacevic A Mavros Y Heisz JJ Fiatarone Singh MA. The effect of resistance exercise on sleep: A systematic review of randomized controlled trials. Sleep Med Rev. (2018) 39:52–68. 10.1016/j.smrv.2017.07.002 28919335

[4] Metcalfe RS Atef H Mackintosh K Mcnarry M Ryde G Hill DM. Time-efficient and computer-guided sprint interval exercise training for improving health in the workplace : a randomised mixed-methods feasibility study in office-based employees. BMC Public Health. (2020) 2020:1–13. 10.1186/s12889-020-8444-z 32164631 PMC7068982

[5] Songsorn P Brick N Fitzpatrick B Fitzpatrick S McDermott G McClean C. Affective and perceptual responses during reduced-exertion high-intensity interval training (REHIT). Int J Sport Exerc Psychol. (2020) 18:717–32. 10.1080/1612197X.2019.159321729172029

[6] Kline CE Sui X Hall MH Youngstedt SD Blair SN Earnest CP. Dose-response effects of exercise training on the subjective sleep quality of postmenopausal women: Exploratory analyses of a randomised controlled trial. BMJ Open. (2012) 2:1–9. 10.1136/bmjopen-2012-001044 22798253 PMC3400065

[7] Ruffino JS Songsorn P Haggett M Edmonds D Robinson AM Thompson D. A comparison of the health benefits of reduced-exertion high-intensity interval training (REHIT) and moderate-intensity walking in type 2 diabetes patients. Appl Physiol Nutr Metab. (2017) 42:202–8. 10.1139/apnm-2016-0497 28121184

[8] Vollaard NBJ Metcalfe RS. Research into the health benefits of sprint interval training should focus on protocols with fewer and shorter sprints. Sport Med. (2017) 47:2443–51. 10.1007/s40279-017-0727-x 28391489 PMC5684281

[9] Metcalfe RS Babraj JA Fawkner SG Vollaard NBJ. Towards the minimal amount of exercise for improving metabolic health: Beneficial effects of reduced-exertion high-intensity interval training. Eur J Appl Physiol. (2012) 112:2767–75. 10.1007/s00421-011-2254-z 22124524

[10] Metcalfe RS Koumanov F Ruffino JS Stokes KA Holman GD Thompson D. Physiological and molecular responses to an acute bout of reduced-exertion high-intensity interval training (REHIT). Eur J Appl Physiol. (2015) 115:2321–34. 10.1007/s00421-015-3217-6 26156806

[11] Gillen JB Percival ME Skelly LE Martin BJ Tan RB Tarnopolsky MA. Three minutes of all-out intermittent exercise per week increases skeletal muscle oxidative capacity and improves cardiometabolic health. PLoS ONE. (2014) 9:e111489. 10.1371/journal.pone.0111489 25365337 PMC4218754

[12] Tobaldini E Costantino G Solbiati M Cogliati C Kara T Nobili L. Sleep, sleep deprivation, autonomic nervous system and cardiovascular diseases. Neurosci Biobehav Rev. (2017) 74:321–9. 10.1016/j.neubiorev.2016.07.004 27397854

[13] Tietjens JR Claman D Kezirian EJ de Marco T Mirzayan A Sadroonri B. Obstructive sleep apnea in cardiovascular disease: A review of the literature and proposed multidisciplinary clinical management strategy. J Am Heart Assoc. (2019) 8:1–17. 10.1161/JAHA.118.010440 30590966 PMC6405725

[14] Bertani RF Campos GO Perseguin DM Bonardi JMT Ferriolli E Moriguti JC. Resistance exercise training is more effective than interval aerobic training in reducing blood pressure during sleep in hypertensive elderly patients. J strength Cond Res. (2018) 32:2085–90. 10.1519/JSC.0000000000002354 29283931

[15] Fornaro M Mccarthy MJ Berardis D De Tabaton M Martino M Colicchio S. Adjunctive agomelatine therapy in the treatment of acute bipolar II depression : a preliminary open label study. Neuropsychiatr Dis Treat. (2013) 9:243–51. 10.2147/NDT.S41557 23430979 PMC3575211

